# Editorial: Combat sports athletes: influence of rapid weight loss on psychological and physiological responses

**DOI:** 10.3389/fpsyg.2025.1579663

**Published:** 2025-03-31

**Authors:** Roberto Roklicer, Barbara Gilic, Tatjana Trivic, Patrik Drid

**Affiliations:** ^1^Faculty of Sport and Physical Education, University of Novi Sad, Novi Sad, Serbia; ^2^Faculty of Kinesiology, University of Split, Split, Croatia

**Keywords:** combat sports, weight reduction, sport psychology, performance, physiology

Rapid weight loss (RWL) is commonly practiced among combat sports athletes who aim to qualify for lower weight categories. Although this strategy may provide a competitive edge, it carries significant physiological and psychological risks. This Research Topic compiles studies that explore the multifaceted effects of RWL on athletes, offering insights into changes in body composition, hydration status, pain sensitivity, psychological resilience, and performance metrics.

Donmez et al. investigated the changes in body composition and urine specific gravity (USG) among elite Turkish Greco-Roman wrestlers. The study involved 31 national team members divided into weight-loss and non-weight-loss groups. Measurements were taken at three time points: the start of training, pre-competition weigh-in, and immediately before the competition. Results indicated that wrestlers who underwent weight reduction experienced significant losses in body mass, fat-free mass, fat mass, and total body water due to dehydration. The athletes only partially recovered during rehydration, so they did not fully regain their optimal condition before the competition, which could have adversely affected their health and performance. The study emphasizes the importance of educating athletes and coaches on safe weight management and proper hydration practices to minimize the negative impacts of RWL.

Çağlar et al. examined the impact of RWL on psychological resilience and mechanical pain sensitivity in elite kickboxers. Thirty-seven male athletes underwent assessments of psychological resilience and pressure pain thresholds (PPT) in the thoracolumbar region before and after a 1-month RWL period before the competition. The study findings revealed significant decrease in PPT values in all measured segments after RWL, indicating increased pain sensitivity. Simultaneously, specific dimensions of psychological resilience, such as structured style and social competence, showed significant improvements, suggesting that structured routines and social support may enhance resilience during RWL periods. This study highlights the complex relationship between physiological stressors and psychological adaptability in combat athletes undergoing RWL.

Demirkan et al. investigated the immediate effects of endurance, strength, and wrestling-specific training on hydration status and performance in young wrestlers. The study found that endurance training led to the most significant acute dehydration and performance declines, with notable reductions in body mass, back strength, grip strength, vertical jump height, and maximal inspiratory pressure. In contrast, strength and wrestling-specific training sessions resulted in less pronounced hydration loss and performance impairments. These findings suggest that endurance exercises, used for rapid weight reduction, may negatively affect hydration and performance more than other training types. The authors recommend strategically scheduling endurance sessions and enhancing hydration strategies to mitigate these acute effects.

Sarıakçalı et al. examined the physiological and psychological consequences of RWL in wrestlers. The study documented that RWL can lead to dehydration, hormonal imbalances, and altered kidney function. Psychologically, athletes undergoing RWL reported increased anxiety, mood disturbances, and disordered eating behaviors. The authors recommend implementing gradual weight loss strategies, regular health monitoring, and providing nutritional and psychological support to reduce the adverse impact of RWL on wrestlers' health and performance.

Yu et al. explored the effects of combined slow and RWL on physiological performance, mood state, and sleep quality in male freestyle wrestlers. The study involved a 30-day slow weight loss phase followed by a 7-day RWL period leading to competition. Findings indicated significant increase in morning heart rate, creatine kinase levels, and fatigue scores during the RWL phase, along with reductions in hemoglobin and testosterone levels. While changes in mood state and sleep quality were not statistically significant, the study underscores the need to carefully monitor physiological parameters during weight management in wrestlers to balance performance and wellbeing.

Garrido-Muñoz et al. explored the relationship between psychological resilience, athletic experience, and competitive level among judokas. This study assessed 702 judokas and found that higher competitive levels and longer participation in judo were associated with increased psychological resilience. Men exhibited significantly higher resilience levels compared to women. These findings suggest that sustained participation and higher competition levels in judo may enhance psychological resilience, emphasizing the importance of experience and competitive exposure in developing mental toughness among combat athletes.

## Conclusions

The collective findings from these studies highlight the critical need for awareness and strategies surrounding the challenges posed by RWL in combat sports. Ensuring the health and performance of athletes requires a comprehensive understanding of both the physiological and psychological factors associated with RWL.

